# The farnesoid X receptor negatively regulates osteoclastogenesis in bone remodeling and pathological bone loss

**DOI:** 10.18632/oncotarget.20576

**Published:** 2017-08-28

**Authors:** Ting Zheng, Ju-Hee Kang, Jung-Sun Sim, Jung-Woo Kim, Jeong-Tae Koh, Chan Soo Shin, Hyungsik Lim, Mijung Yim

**Affiliations:** ^1^ College of Pharmacy, Sookmyung Women's University, Yongsan-ku, Seoul, Republic of Korea; ^2^ Department of Pharmacology and Dental Therapeutics, Research Center for Biomineralization Disorders, School of Dentistry, Chonnam National University, Gwangju, Republic of Korea; ^3^ Department of Internal Medicine, College of Medicine, Seoul National University, Seoul, Republic of Korea; ^4^ Departments of Physics, Hunter College of the City University of New York, New York City, New York, USA

**Keywords:** FXR, osteoclast, RANKL, NFATc1, bone loss

## Abstract

Farnesoid X receptor (FXR, *NR1H4*) is a member of the nuclear receptor superfamily of ligand-activated transcription factors. Since the role of FXR in osteoclast differentiation remains ill-defined, we investigated the biological function of FXR on osteoclastogenesis, using FXR-deficient mice. We demonstrated that FXR deficiency increases osteoclast formation *in vitro* and *in vivo*. First, FXR deficiency was found to accelerate osteoclast formation via down-regulation of c-Jun N-terminal kinase (JNK) 1/2 expression. Increased expression of peroxisome proliferator-activated receptor (PPAR)γ and peroxisome proliferator-activated receptor gamma coactivator 1 (PGC-1)β seems to mediate the pro-osteoclastogenic effect of FXR deficiency via the JNK pathway. In addition, we found that FXR deficiency downregulated the expression of interferon-β (IFN-β), a strong inhibitor of osteoclastogenesis, via receptor activator of nuclear factor-kappaB ligand (RANKL). We further suggested that interference of IFN-β expression by FXR deficiency impaired the downstream JAK3-STAT1 signaling pathways, which in turn increased osteoclast formation. Finally, FXR deficiency accelerated unloading- or ovariectomy-induced bone loss *in vivo*. Thus, our findings demonstrate that FXR is a negative modulator in osteoclast differentiation and identify FXR as a potential therapeutic target for postmenopausal osteoporosis and unloading-induced bone loss.

## INTRODUCTION

Bone is consistently renewed throughout life by the opposing activities of osteoblastic bone formation and osteoclastic bone resorption pathways. Although osteoclasts are required for bone remodeling, excess activity of osteoclasts can lead to various diseases such as periodontal disease, osteoporosis, rheumatoid arthritis, multiple myeloma, and metastatic cancers [1–3]. Osteoclasts are giant multinucleated cells derived from hematopoietic precursors of the monocyte-macrophage lineage. During osteoclastogenesis, bone marrow-derived macrophages (BMMs) differentiate into tartrate-resistant acid phosphatase (TRAP)-positive pre-osteoclasts, which then fuse with each other to form mature osteoclasts [4–6].

Macrophage colony-stimulating factor (M-CSF) and receptor activator of nuclear factor kappa-B ligand (RANKL) provide the two necessary and sufficient signals for osteoclast differentiation. Binding of M-CSF to its receptor, CSF-1R, in osteoclast precursors promotes their proliferation and survival via the activation of kinases such as Src, phospholipase C-gamma (PLC-γ), phosphoinositide 3-kinase (PI_3_K), protein kinase B (PKB, also AKT) and extracellular signal-regulated kinase 1/2 (ERK1/2) [7–9]. RANKL binding to RANK induces the association of RANK with tumor necrosis factor receptor-associated factor 6 (TRAF6), which activates nuclear factor-kappaB (NF-κB) and mitogen-activated protein kinases (MAPKs; ERK1/2, p38, and stress-activated protein kinase/c-Jun N-terminal kinase (SAPK/JNK)). In turn, these kinases activate nuclear factor of activated T cells (NFATc1), the master transcription factor responsible for osteoclast differentiation and function [10].

The farnesoid X receptor (FXR, *NR1H4*) is a member of the nuclear receptor superfamily of transcription factors that regulates multiple biological processes. Bile acid has been identified as endogenous ligands for FXR [11–13]. Upon ligand-induced activation, FXR binds to FXR response elements [14] either as a monomer or as a heterodimer with the retinoid X receptor (RXR) [13, 14] and modulates bile acids, lipid and glucose metabolism, inflammation, and energy metabolism [11, 12]. Given all of these benefits, FXR has been considered as a therapeutic target for the treatment of metabolic disorders [19, 20].

Several studies suggest that FXR has complex roles in the pathogenesis of metabolic dysfunction, and its activity in different tissues could possibly exert different effects on metabolism [11–20]. We previously demonstrated that the deletion of FXR (FXR*^−/−^*) *in vivo* resulted in a significant reduction in bone mineral density compared with that in FXR^+/+^ mice [21]. Since the mechanism by which FXR modulates bone metabolism has not been fully explored, we investigated a specific role of FXR in the modulation of osteoclastogenesis and bone loss *in vitro* and *in vivo*. Our work demonstrates that FXR could be exploited to prevent the development of osteoclast-associated bone diseases.

## RESULTS

### FXR deficiency facilitates osteoclast differentiation

**Figure 1:**
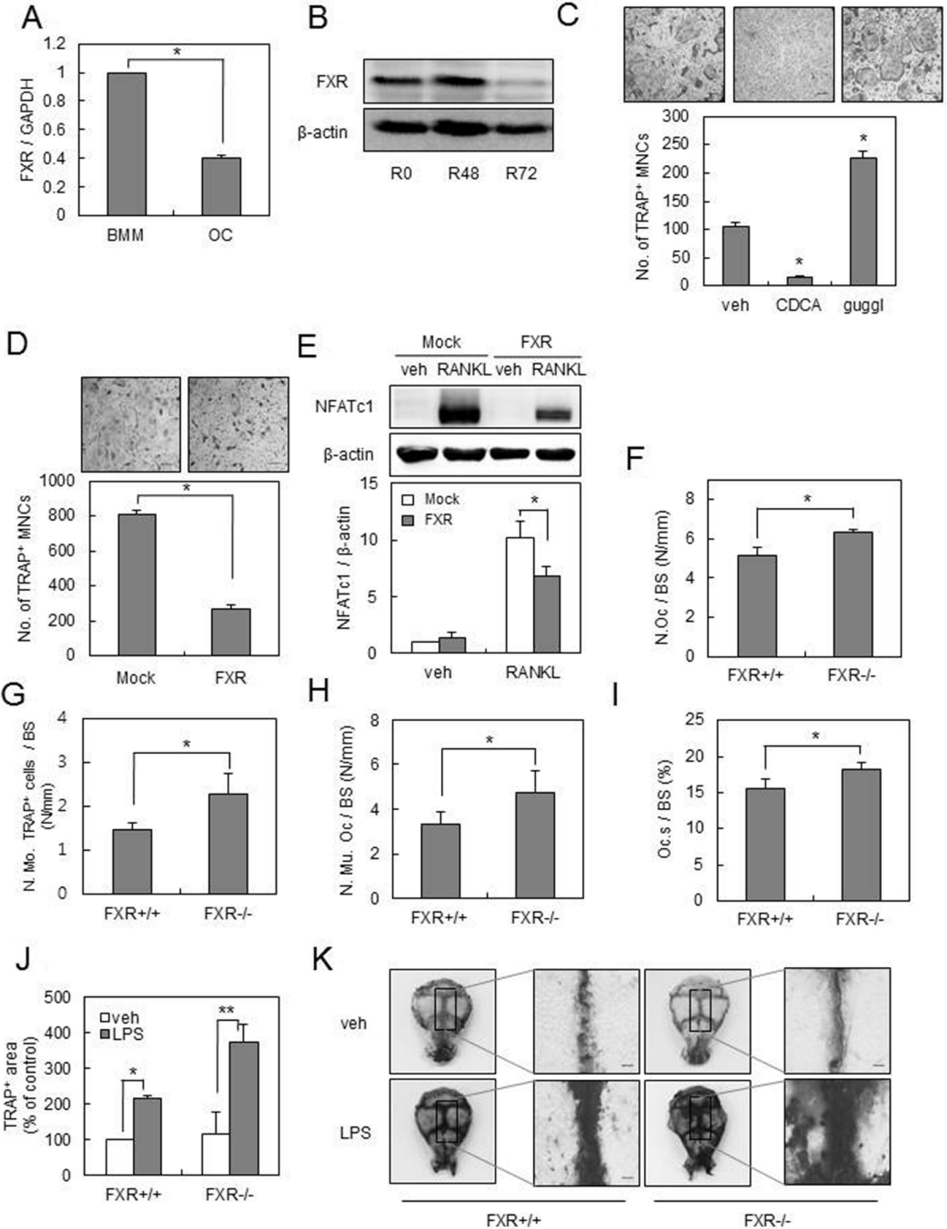
FXR negatively regulates osteoclast formation (**A**) BMMs from 12-week-old mice were cultured with RANKL (100 ng/ml) and M-CSF (30 ng/ml) for 4 days. The mRNA levels of FXR were analyzed by real-time PCR. (**B**) BMMs from 12-week-old mice were cultured with RANKL (100 ng/ml) for the indicated times and FXR expression levels were analyzed by western blot. (**C**) BMMs were cultured with RANKL (100 ng/ml) and M-CSF (30 ng/ml) in the presence of 75 μM CDCA or 0.3 μM guggulsterone for 4 days. TRAP^+^ MNCs were counted as osteoclasts when more than 3 nuclei were present. Scale bar, 200 μm. (**D**) BMMs were infected by mock or FXR through a retrovirus packaging system. Infected BMMs were cultured with RANKL (100 ng/ml) and M-CSF (30 ng/ml) for 4 days. Scale bar, 200 μm. (**E**) Infected BMMs were cultured with M-CSF (30 ng/ml) in the presence or absence of RANKL (200 ng/ml) for 48 h. Cell lysates were then subjected to western blot analysis with anti-NFATc1 antibody. (**F**–**I**) Histological bone sections from distal femur of FXR^+/+^ and FXR*^−/−^*mice were analyzed. N.Oc./BS (F), N.Mo. TRAP^+^ cells/BS (G), N.Mu.Oc/BS (H) and Oc.S/BS (I). *n* = 6 for each group. (**J**–**K**) Calvarias of FXR^+/+^ and FXR*^−/−^* mice that received the vehicle or LPS were subjected to TRAP staining. TRAP^+^ stained area in calvaria was quantified using ImageJ. Representative images are shown in (K). *n* = 5 for each group. Scale bar, 500 μm. *p < 0.05, **p < 0.01. N.Oc./BS: osteoclast number/bone surface; N.Mo.TRAP^+^ cells/BS: number of TRAP^+^ mononuclear cells/bone surface; N.Mu.Oc/BS: number of multinuclear osteoclast/bone surface; Oc.S/BS: osteoclast surface/bone surface.

To explore the functional role of FXR in osteoclast differentiation, we first examined the endogenous expression of FXR in BMMs by RANKL. The mRNA and protein expression levels of FXR were significantly decreased in BMMs by RANKL treatment (Figure 1A, 1B). Thus, we investigated the role of FXR on RANKL-induced osteoclast formation, using an activator and an inhibitor of FXR. Activation of FXR by chenodeoxycholic acid (CDCA) decreased the formation of TRAP^+^ multinucleated cells (MNCs), whereas inhibition of FXR by guggulsterone significantly enhanced the formation of TRAP^+^ MNCs by RANKL (Figure 1C). We next overexpressed FXR in BMMs, using a retrovirus (Supplementary Figure 1). Overexpression of FXR in BMMs significantly inhibited the formation of TRAP^+^ MNCs mediated by RANKL when compared with that in the control vector (Figure 1D). In accordance with these results, overexpression of FXR attenuated the RANKL-induced expression of NFATc1 during RANKL-mediated osteoclastogenesis (Figure 1E). Taken together, these data suggested that FXR has a negative role in RANKL-mediated osteoclast differentiation.

To further investigate the role of FXR on osteoclast formation *in vivo*, we conducted histomorphometric analyses of bone sections from the distal femur of FXR^+/+^ and *FXR^−/−^* mice. *FXR* deficiency increased the number of osteoclasts per bone surface (N.Oc/BS; Figure 1F). Both the number of TRAP^+^ mononuclear cells (N.Mo.TRAP^+^ cells/BS) and multinuclear osteoclasts (N.Mu.Oc/BS) per bone surface were increased in *FXR^−/−^* compared with *FXR*^+/+^ femur (Figure 1G and 1H). *FXR* deficiency also enhanced the levels of osteoclast surface per bone surface (Oc.S/BS; Figure 1I). These observations indicate that FXR deficiency increases osteoclastogenesis *in vivo*. Furthermore, the role of FXR in the context of inflammation-induced osteoclastogenesis was examined using the lipopolysaccharide (LPS)-challenged bone loss model. LPS stimulates osteoclast formation *in vivo*. When LPS was injected into the supra calvaria region of mice, the number of TRAP stained-osteoclasts was markedly increased in the calvaria of *FXR^−/−^* compared with that of FXR^+/+^ mice (Figure 1J, 1K). Collectively, these results suggest that *FXR* is a crucial negative regulator of osteoclast differentiation.

### FXR deficiency accelerates osteoclast formation via downregulation of JNK 1/2 expression

**Figure 2:**
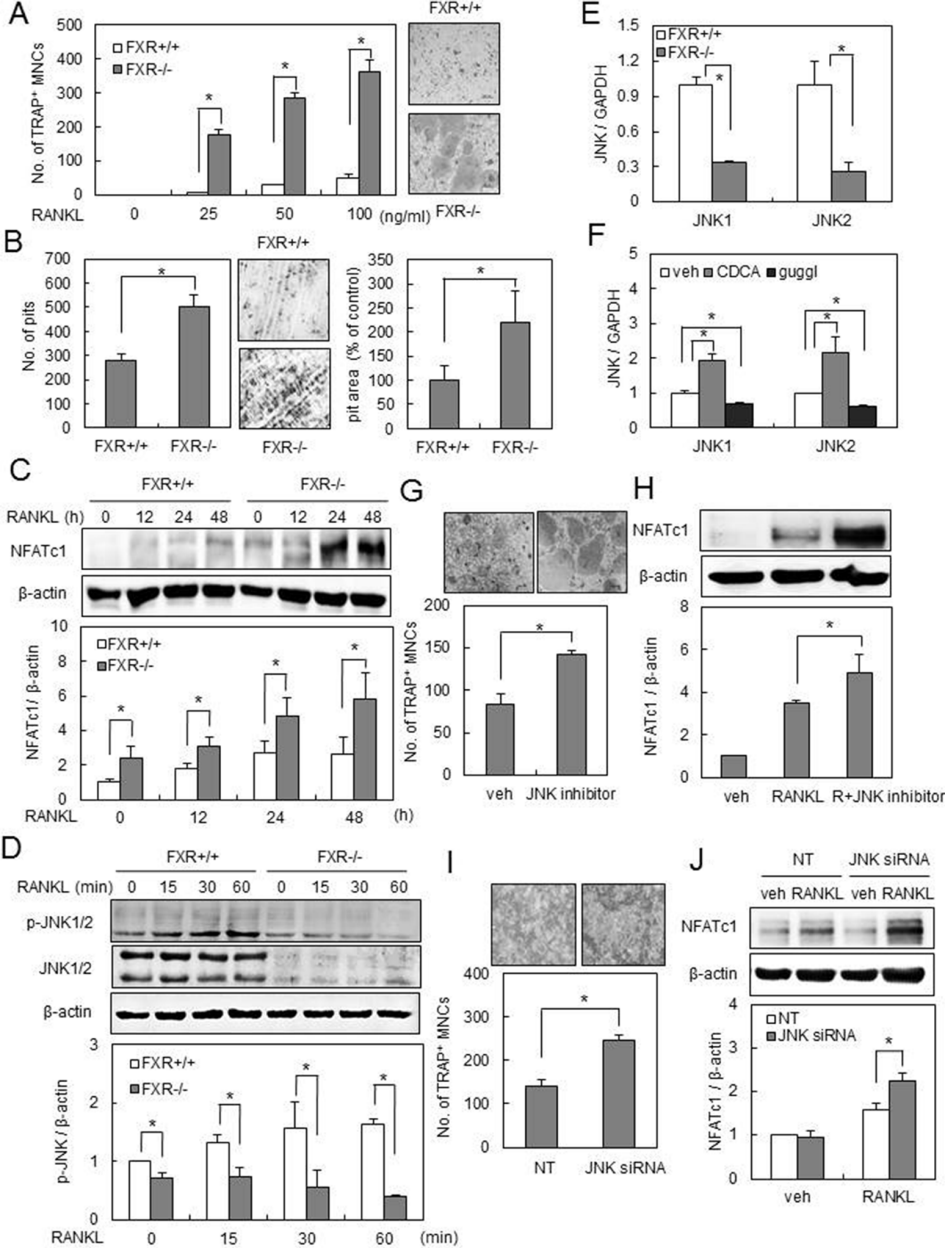
FXR deficiency increases osteoclast differentiation via downregulation of JNK1/2 expression (**A**) BMMs from FXR^+/+^ and FXR*^−/−^* mice were cultured with M-CSF and the indicated concentration of RANKL for 4 days, and TRAP^+^ osteoclasts were counted. (**B**) BMMs from FXR^+/+^ and FXR*^−/−^* mice were placed on dentine slices and cultured in the presence of RANKL (100 ng/ml) for 6 days. The remaining cells were removed and stained with toluidine blue. The images were observed under the microscope. The number of resorbed pits was counted and pit area in each dentine was quantified using ImageJ. (**C**) BMMs from FXR^+/+^ and FXR*^−/−^*mice were cultured with RANKL (200 ng/ml) for the indicated time. (**D**) BMMs from FXR^+/+^ and FXR*^−/−^* mice were serum-starved for 16 h and stimulated with RANKL (200 ng/ml) for the indicated time. Cell lysates were then subjected to western blot analysis with anti-NFATc1, anti-p-JNK, or anti-JNK antibody. (**E**) The mRNA expression of JNK1 and JNK2 in FXR^+/+^ and FXR*^−/−^* BMMs was analyzed by real-time PCR. (**F**) FXR^+/+^ BMMs were cultured in the presence of 75 μM CDCA or 0.3 μM guggulsterone for 48 h. The mRNA expression of JNK1 and JNK2 was analyzed by real-time PCR. (**G**) FXR^+/+^ BMMs were cultured with RANKL (100 ng/ml) in the presence or absence of SP600125 (0.3 μM), a JNK inhibitor, for 4 days. (**H**) FXR^+/+^ BMMs were cultured with RANKL (200 ng/ml) in the presence or absence of SP600125 (0.3 μM) for 48 h. Cell lysates were then subjected to western blot analysis with anti-NFATc1 antibody. (**I**) FXR^+/+^ BMMs were transfected with 40 nM siRNA. The siRNA-transfected FXR^+/+^ BMMs were cultured with RANKL (200 ng/ml) for 3 days, and then TRAP^+^ osteoclasts were counted. (**J**) The siRNA-transfected FXR^+/+^ BMMs were cultured with RANKL (200 ng/ml) for 48 h. Cell lysates were then subjected to western blot analysis with anti-NFATc1 antibody. Data are expressed as means ± SD from at least three independent experiments. Scale bar, 200 μm. *p < 0.05.

To clarify the mechanism of increased osteoclastogenesis by FXR deletion, we performed *in vitro* experiments, using BMMs isolated from *FXR^−/−^ and FXR*^+/+^ mice. We first showed that *FXR^−/−^* BMMs exhibited more TRAP^+^ MNCs by RANKL in a dose-dependent manner, in comparison to FXR^+/+^ BMMs (Figure 2A). In addition, when cultured on dentine slices with RANKL, *FXR−/−* BMMs generated more resorption pits than FXR^+/+^ BMMs, with larger overall area (Figure 2B).

To examine the mechanisms underlying the osteoclastogenic increase in *FXR^−/−^* BMMs, we investigated the expression of NFATc1, a key regulator of osteoclast differentiation. There was a higher increase in the expression level of NFATc1 by RANKL in *FXR−/−* BMMs than in FXR^+/+^ BMMs (Figure 2C). Next, we investigated the role of *FXR* in the regulation of RANKL-dependent signaling pathways, including ERK, p38, JNK1/2, NF-κB, and AKT. There was no significant difference in ERK, p38, NF-κB, and AKT activation by RANKL between FXR^+/+^ and *FXR−/−* BMMs (data not shown). In contrast, JNK1/2 phosphorylation by RANKL was suppressed in *FXR^−/−^* BMMs with a substantial change in protein expression (Figure 2D). The reduction of JNK1/2 protein expression in *FXR^−/−^* BMMs was due to decreased JNK1/2 mRNA levels (Figure 2E). Modulation of JNK1/2 expression levels was further confirmed by pharmacological activation (CDCA) or inhibition (guggulsterone) of FXR (Figure 2F), and by overexpression of FXR (Supplementary Figure 2). Paradoxically, JNK is known to be critical for RANKL-stimulated NFATc1 induction and osteoclast formation [2–4]. Thus, we investigated whether pharmacological JNK inhibition by SP600125 or down-regulation of JNK by siRNA would closely mimic the genetic deletion of FXR. Since the basal level of JNK1/2 phosphorylation was maintained in *FXR^−/−^* BMMs by RANKL (Figure 2D), we used a lower concentration (0.3 μM) of SP600125 than is normally used (1–10 μM). Interestingly, a low level of JNK1/2 inhibition led to a strong increase in osteoclastic differentiation by RANKL accompanied by a significant induction of NFATc1 (Figure 2G, 2H). Similar effects were seen by the use of siRNAs. The introduction of JNK-specific siRNA into FXR^+/+^ BMMs showed a similar reduction in JNK1/2 mRNA expression similar to that in *FXR^−/−^* BMMs (Supplementary Figure 3). Compared to that with the control siRNA, the knock-down of JNK1/2 resulted in a significant increase in NFATc1 expression as well as in the formation of TRAP^+^ MNCs by RANKL (Figure 2I, 2J). These results indicate that *FXR* has a negative role in osteoclast differentiation by modulating JNK signals downstream to RANKL.

### FXR deficiency up-regulates PPARγ and PGC-1β expression via the JNK pathway

**Figure 3:**
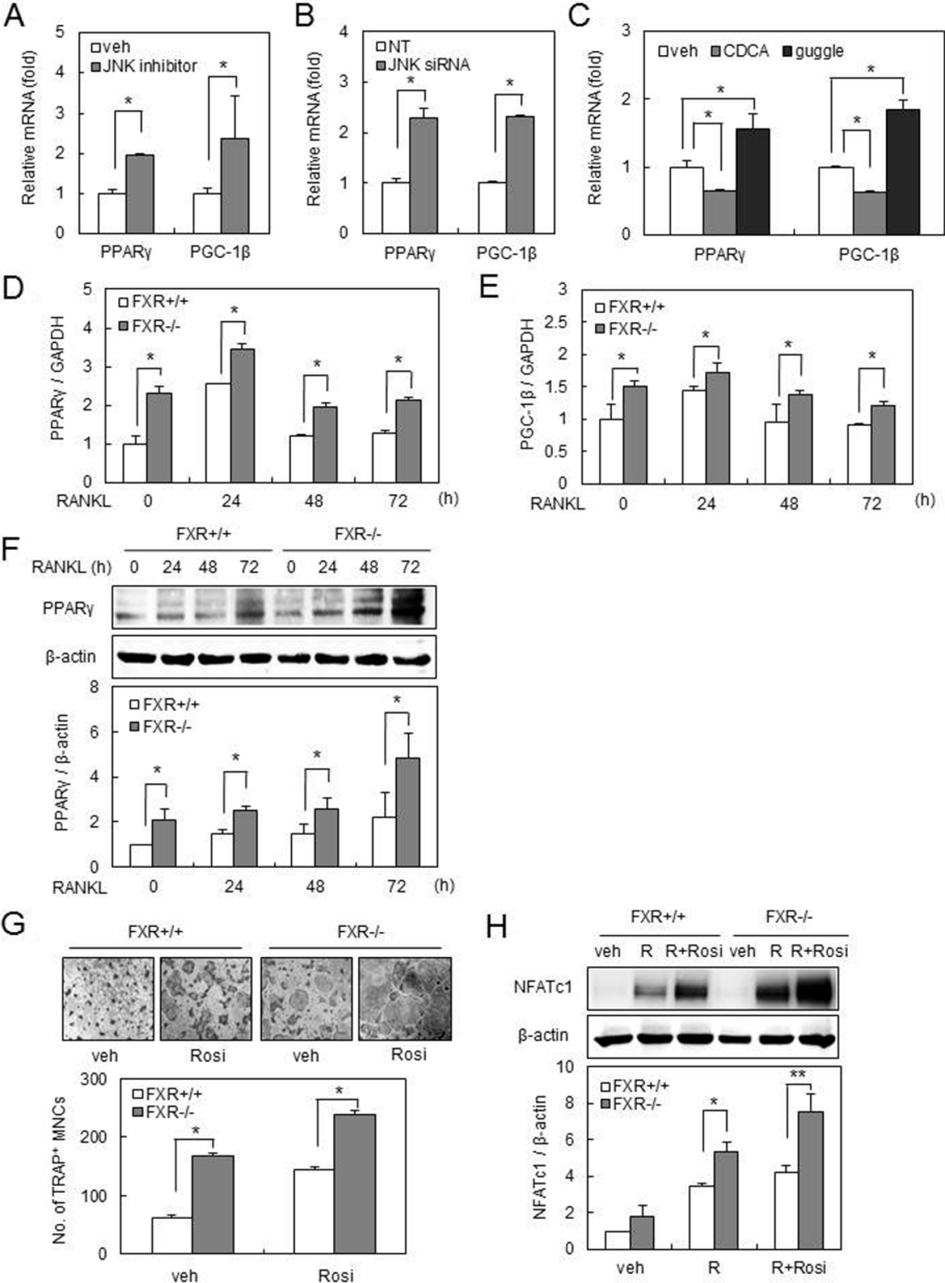
FXR deficiency up-regulates PPARγ and PGC-1β expression via downregulation of JNK (**A**) FXR^+/+^ BMMs were cultured in the presence or absence of SP600125 (0.3 μM) for 24 h. The mRNA expression of PPARγ or PGC-1β was analyzed by real-time PCR. (**B**) FXR^+/+^ BMMs were transfected with 40 nM siRNA. The mRNA expression was analyzed by real-time PCR using PPARγ or PGC-1β primer. (**C**) FXR^+/+^ BMMs were cultured with CDCA (75 μM) or guggulsterone (0.3 μM) for 24 h. The mRNA expression of PPARγ or PGC-1β was analyzed by real-time PCR. (**D**–**E**) BMMs from FXR^+/+^ and FXR*^−/−^* mice were cultured with RANKL (200 ng/ml) for the indicated time. The mRNA level was analyzed by real-time PCR with PPARγ (D) or PGC-1β (E) primer. (**F**) FXR^+/+^ and FXR*^−/−^* BMMs were cultured with RANKL (200 ng/ml) for the indicated time. Cell lysates were then subjected to western blot analysis with anti-PPARγ antibody. (**G**) FXR^+/+^ and FXR*^−/−^* BMMs were cultured with RANKL (100 ng/ml) in the presence or absence of rosiglitazone (0.3 μM) for 3 days. Scale bar, 200 μm. (**H**) BMMs from FXR^+/+^ and FXR*^−/−^* mice were cultured with RANKL (200 ng/ml) in presence or absence of rosiglitazone (0.3 μM) for 48 h. Cell lysates were then subjected to western blot analysis with anti-NFATc1 antibody. Data are expressed as means ± SD from at least three independent experiments. *p < 0.05, **p < 0.01.

To gain insight into how FXR modulates osteoclast formation via JNK, we investigated the expression of genes known to have a role in osteoclastogenesis. We found that JNK inhibitor (0.3 μM) or JNK-specific siRNA enhanced the mRNA expression of peroxisome proliferator-activated receptor (PPAR)γ and peroxisome proliferator-activated receptor-gamma coactivator 1 (PGC-1) β in BMMs (Figure 3A, 3B). Furthermore, activation of FXR by CDCA decreased the mRNA expression of PPARγ and PGC-1β, whereas inhibition of FXR by guggulsterone conversely resulted in an increase in these mRNA expressions (Figure 3C). Real-time PCR demonstrated that the mRNA expression of PPARγ and PGC-1β was significantly increased in FXR*^−/−^* BMMs compared with that in FXR^+/+^ BMMs, which is consistent with these results (Figure 3D, 3E). Moreover, RANKL-induced mRNA expression levels of PPARγ and PGC-1β were maintained at significantly higher levels in FXR*^−/−^* BMMs (Figure 3D, 3E). Similarly, the up-regulation of PPARγ protein expression in FXR*^−/−^* BMMs was confirmed by western blot (Figure 3F).

PPARγ is a member of the nuclear hormone receptor superfamily of ligand-responsive transcription factors that can be activated by lipophilic ligands. PPARγ activation by rosiglitazone promotes hematopoietic stem cell (HSC) differentiation into osteoclasts, thus increasing bone resorption [24, 25]. PGC-1β is a transcriptional coactivator that regulates energy metabolism by stimulating mitochondrial biogenesis and respiration of cells [26–29]. PGC-1β is required for the pro-osteoclastogenic and bone resorption-enhancing effects of PPARγ [24].

Since *FXR^−/−^* BMMs have higher expression levels of PPARγ and PGC-1β compared to FXR^+/+^ BMMs, we investigated the effect of rosiglitazone, a PPARγ agonist, on RANKL-induced osteoclast differentiation from BMM cells. As previously reported [30], rosiglitazone increased osteoclast formation from FXR^+/+^ BMMs, and this induction was accelerated in *FXR^−/−^* BMMs (Figure 3G). We also revealed that the level of *NFATc1* was increased by rosiglitazone in *FXR+/+* cells, but was significantly higher in *FXR^−/−^* cells (Figure 3H). These data suggest that up-regulation of PPARγ and PGC-1β expression by suppression of the JNK pathway leads to the increased osteoclast formation in *FXR^−/−^* BMMs.

### FXR deficiency accelerates rosiglitazone-induced bone loss *in vivo*

**Figure 4:**
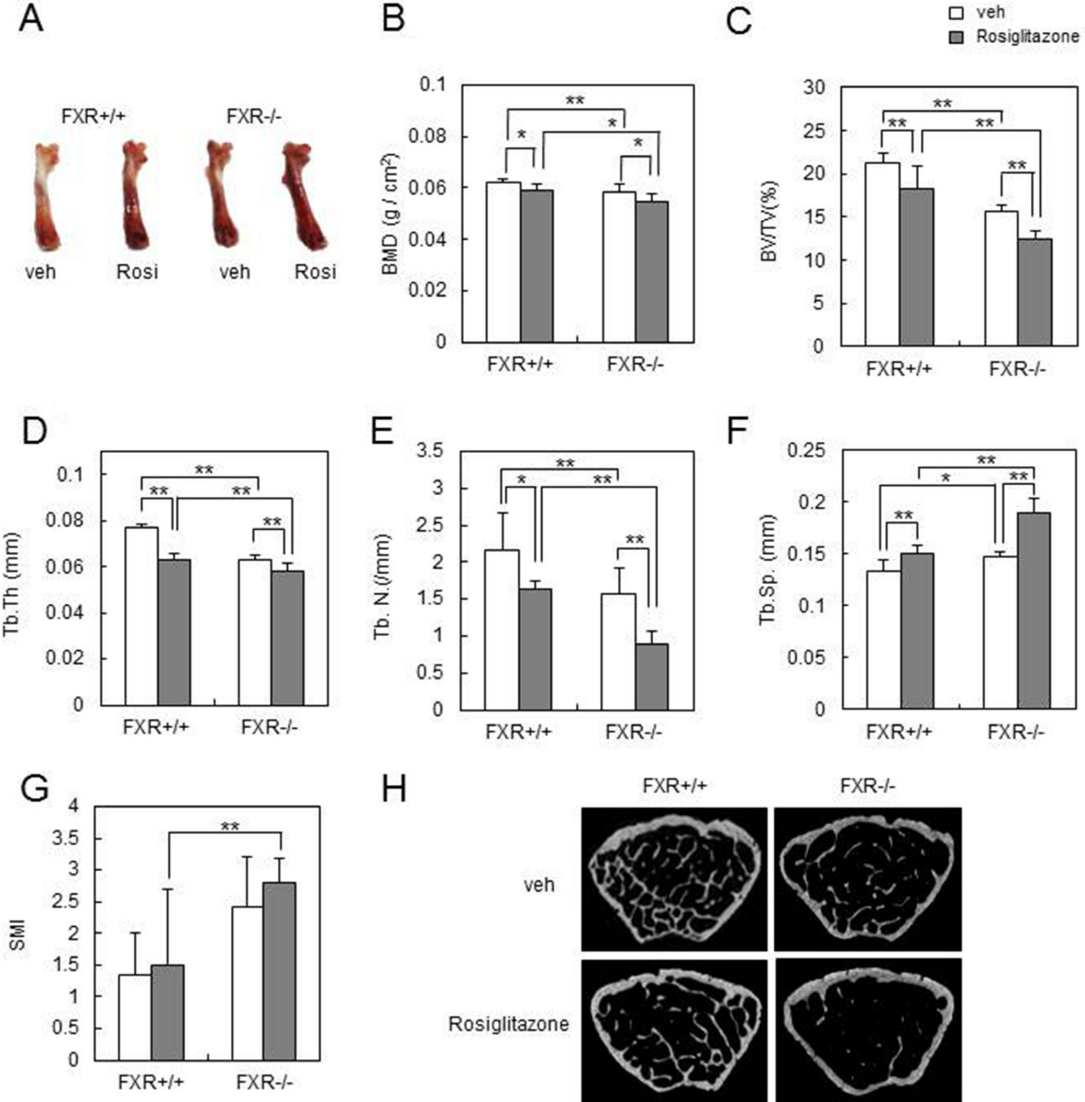
FXR deficiency increases rosiglitazone-induced bone loss *in vivo* Male 12-week-old FXR^+/+^ and FXR*^−/−^* mice were orally administered daily with rosiglitazone (10 mg/kg) for 2 months, and mouse femurs were collected. (**A**) Femurs of FXR^+/+^ or FXR*^−/−^* mice from each group. (**B**) BMD (g/cm^2^) of femurs from FXR^+/+^ and FXR*^−/−^* mice was analyzed by DXA. (**C**–**G**) Various bone parameters were analyzed by micro-CT. BV/TV (%) (C), Tb.Th (mm) (D), Tb.N (/mm) (E), Tb.Sp (mm) (F), SMI (G). (H) Two-dimensional micro-CT images of the distal metaphysis of the femurs. *p < 0.05, **p < 0.01. BMD, bone mineral density; BV/TV, bone volume per tissue volume; Tb.Th, trabecular thickness; Tb.N, trabecular number; Tb.Sp, trabecular separation; SMI, structural model index.

To further delineate the effect of increased PPARγ and PGC-1β expression in *FXR^−/−^* BMMs on bone resorption and bone loss *in vivo*, we orally administrated FXR^+/+^ or *FXR^−/−^* mice with rosiglitazone or vehicle at 10 mg/kg/d for 8 weeks. Femurs from the rosiglitazone group became darker in *FXR^−/−^* mice than in FXR^+/+^ mice (Figure 4A). The DXA analysis indicated that rosiglitazone-mediated reduction in BMD was significantly accelerated in the femurs of *FXR^−/−^* mice (Figure 4B). A micro-CT analysis of the metaphyseal region of the femur showed that the trabecular bone volume per tissue volume (BV/TV) was significantly lower in *FXR^−/−^* than FXR^+/+^ mice following rosiglitazone treatment (Figure 4C). Consistently, two other bone parameters related to BV/TV – trabecular thickness (Tb.Th) and trabecular number (Tb.N; linear density of trabecular bone) – were more significantly reduced in FXR*^−/−^* mice compared with FXR^+/+^ mice by rosiglitazone treatment (Figure 4D, 4E). No obvious difference was seen in trabecular separation (Tb.Sp) between rosiglitazone-treated FXR^+/+^ and *FXR^−/−^* mice (Figure 4F). Rosiglitazone treatment also resulted in a high structure model index (SMI) in FXR^+/+^ mice, an indicator of increased fragility, which was more significantly increased in *FXR^−/−^* mice (Figure 4G). Finally, two-dimensional visualization of the femoral area clearly showed that the massive loss of trabecular bone following rosiglitazone treatment was much higher in *FXR^−/−^* mice compared with FXR^+/+^ mice (Figure 4H). These results indicate that FXR deficiency confers sensitivity to rosiglitazone-induced bone resorption and bone loss *in vivo*.

### FXR deficiency accelerates osteoclast formation via downregulation of IFN-β signaling pathways

**Figure 5:**
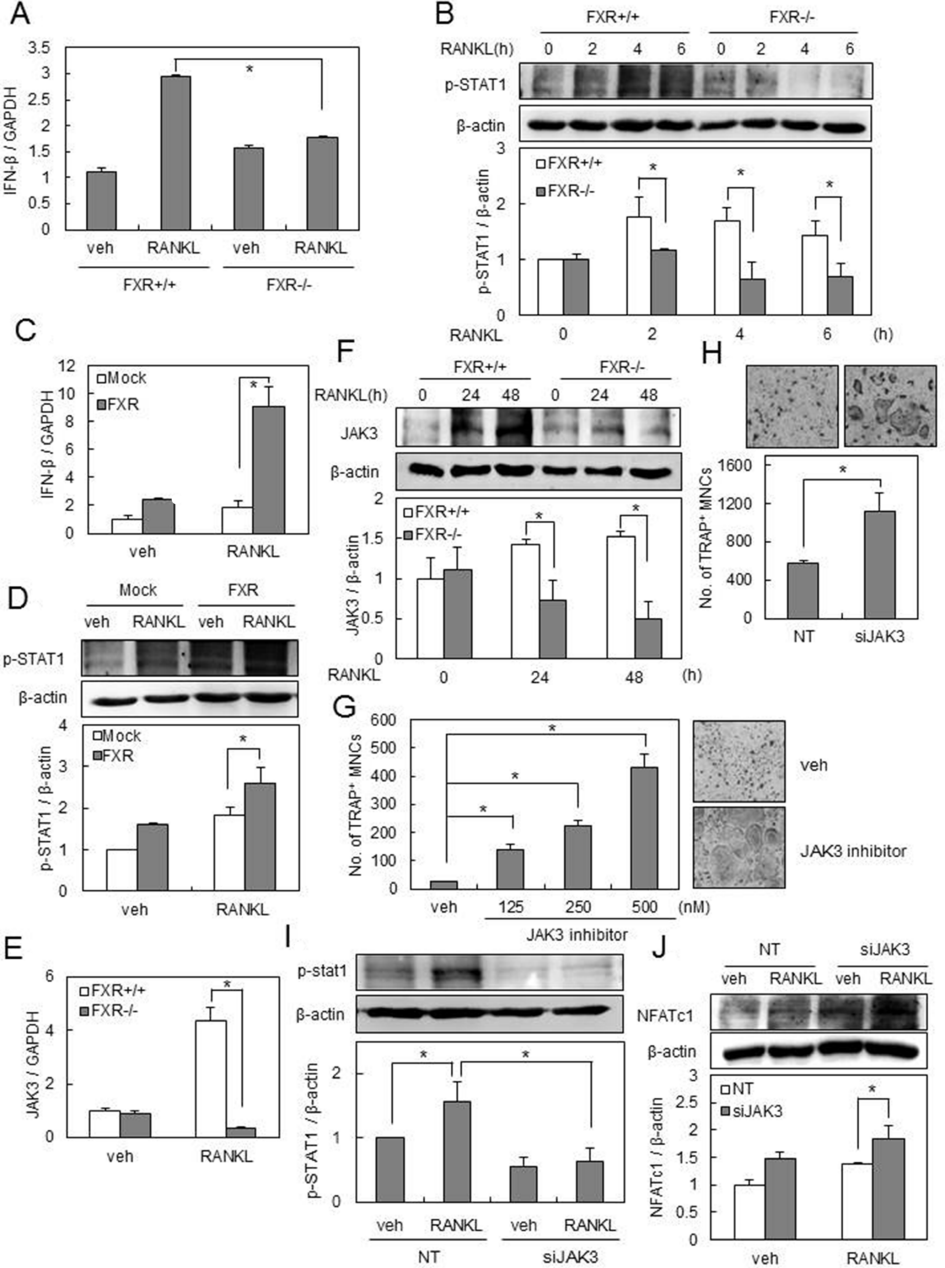
FXR deficiency down-regulates IFN-β signaling pathways via JAK3-STAT1 (**A**, **E**) BMMs from FXR^+/+^ and FXR*^−/−^* mice were cultured with RANKL (200 ng/ml) for 24 h. The mRNA level was analyzed by real-time PCR with IFN-β or JAK3 primer. (**B**) BMMs from FXR^+/+^ and FXR*^−/−^* mice were serum-starved for 16 h and stimulated with RANKL (200 ng/ml) for the indicated time. (**F**) BMMs from FXR^+/+^ and FXR*^−/−^* mice were cultured with RANKL (200 ng/ml) for the indicated time. Cell lysates were then subjected to western blot analysis with anti-p-STAT1 or anti-JAK3 antibody. (**C**, **D**) BMMs were infected by mock or FXR through a retrovirus packaging system. Infected BMMs were stimulated with RANKL (200 ng/ml) for 4 or 24 h. The mRNA levels of IFN-β and p-STAT1 were analyzed by real-time PCR or western blotting. (**G**) BMMs from FXR^+/+^ mice were cultured with RANKL (100 ng/ml) in the presence of tofacitinib, a JAK3 inhibitor, for 3 days. TRAP^+^ MNCs were counted as osteoclasts when more than 3 nuclei were present. Scale bar, 200 μm. (**H**) FXR^+/+^ BMMs were transfected with 40 nM siRNA. The siRNA-transfected FXR^+/+^ BMMs were cultured with RANKL (200 ng/ml) for 3 days, and then TRAP^+^ osteoclasts were counted. (**I**) The siRNA-transfected FXR^+/+^ BMMs were serum-starved for 16 h and stimulated with RANKL (200 ng/ml) for 4 h. (**J**) The siRNA-transfected FXR^+/+^ BMMs were cultured with RANKL (200 ng/ml) for 24 h. Cell lysates were then subjected to western blotting analysis with anti-p-STAT1 or anti-NFATc1 antibody. Data are expressed as mean ± SD from at least three independent experiments. Scale bar, 200 μm. *p < 0.05.

To balance out osteoclastogenesis, RANKL also induces the *IFN-β* gene in BMMs, and this induction constitutes a critical aspect of the negative feedback regulation of RANKL signaling that allows for the suppression of excessive osteoclastogenesis [31]. Since FXR deficiency was shown to increase the pro-osteoclastogenic signals from RANKL, we next investigated whether FXR deficiency modulates the anti-osteoclastogenic mechanism of RANKL. RANKL increased mRNA expression of IFN-β in FXR^+/+^ BMMs, and this induction was significantly decreased in *FXR−/−* BMMs (Figure 5A). The phosphorylation of STAT1 is known to be essential for IFN-β signaling by RANKL [31]. The level of p-STAT1 by RANKL was increased in *FXR*^+/+^ BMMs, which was completely abrogated in *FXR^−/−^* BMMs (Figure 5B). In accord with these findings, overexpression of FXR in BMMs increased the level of IFN-β and p-STAT1 by RANKL when compared with the results with control vector (Figure 5C, 5D). The STAT family is located downstream of the JAKs [30]. We further demonstrated that RANKL increases JAK3 mRNA and protein levels in FXR^+/+^ BMMs, but not in *FXR^−/−^* BMMs (Figure 5E, 5F).

Since the role of JAK3 in osteoclast formation has not been determined [32], we next assessed the effect of JAK3 downregulation on osteoclast differentiation. We found that a pharmacological JAK3 inhibitor accelerates RANKL-induced osteoclastogenesis in a dose-dependent manner (Figure 5G). Similar effects were seen in BMMs transfected with JAK3-specific siRNA (Figure 5H). In addition, compared with the control, knock-down of JAK3 in BMMs resulted in a significant decrease in the level of p-STAT1 (Figure 5I), followed by increased NFATc1 expression by RANKL (Figure 5J). These data suggest that the impairment of IFN-β pathways via JAK3-STAT1 is another mechanism underlying the increased osteoclast formation in *FXR^−/−^* BMMs.

### FXR deficiency accelerates unloading- or OVX-induced bone loss *in vivo*

We showed that FXR has an important role in OC differentiation and bone remodeling under physiological conditions. Next we used an unloading- or OVX-induced bone loss model in order to determine whether deletion of *FXR* affects bone loss under pathological conditions.

**Figure 6:**
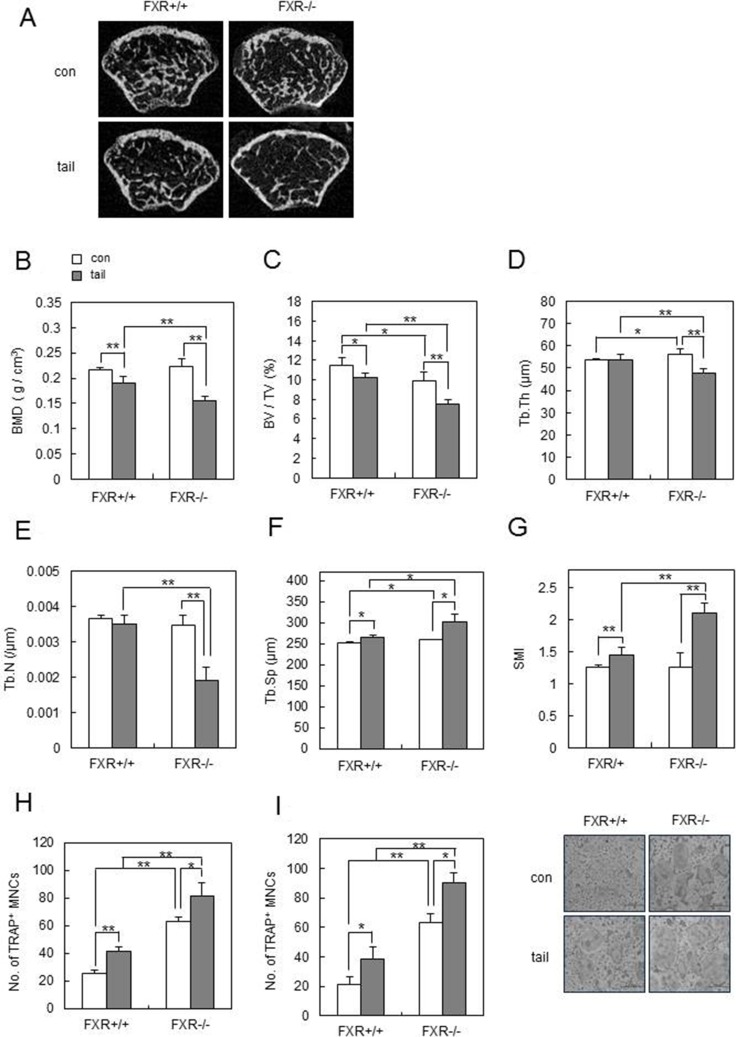
FXR deficiency accelerates unloading-induced bone loss *in vivo* Male 8-week-old FXR^+/+^ and FXR*^−/−^* mice were subjected to tail suspension, and mouse femurs were collected after 1 week. (**A**) Two-dimensional micro-CT images of the distal metaphysis of the femurs. (**B**–**G**) Various bone parameters of femurs were analyzed by micro-CT. BMD (g/cm^3^) (B), BV/TV (%) (C), Tb.Th (μm) (D), Tb.N (/μm) (E), Tb.Sp (μm) (F), SMI (G). (**H**–**I**) bone marrow cells (H) or BMMs (I) from FXR^+/+^ and FXR*^−/−^* mice were cultured with RANKL (100 ng/ml) and M-CSF (30 ng/ml) for 4 days and then TRAP^+^ osteoclasts were counted. *p < 0.05, **p < 0.01.

The effect of unloading was first investigated in FXR-deficient mice, using tail suspension as a model of hindlimb unloading [33]. FXR^+/+^ and FXR^−/−^ mice were subjected to tail suspension at age 8 weeks, and their skeletal response was analyzed 1 week later. Unloading induced a significantly greater femoral bone loss in *FXR*^–/–^ mice than in FXR^+/+^ mice (Figure 6A). In agreement, BMD was significantly lower in *FXR*^–/–^ mice than in FXR^+/+^ mice after hindlimb unloading (Figure 6B). As FXR deficiency accelerates the unloading-induced reduction in BMD levels, we next examined the effects FXR deficiency on the microstructural bone deterioration that is induced by hindlimb unloading. Three dimensional micro-CT analyses revealed that the level of BV/TV in the metaphyseal region of the femur was reduced by hindlimb unloading in FXR^+/+^ mice, and FXR deficiency significantly increased the unloading-induced reduction in BV/TV (Figure 6C). Other bone parameters, including Tb.Th, Tb.N, Tb,Sp, and SMI, also confirmed more severe bone loss by hindlimb unloading in *FXR^−/−^* mice compared to FXR^+/+^ mice (Figure 6D–6G). Furthermore, osteoclastogenesis was increased in bone marrow (Figure 6H) or BMM cells (Figure 6I) from FXR^-/-^ mice, in comparison to those from FXR^+/+^ mice after tail-suspension. These data demonstrated that FXR deficiency accelerates unloading-induced bone loss *in vivo*.

**Figure 7:**
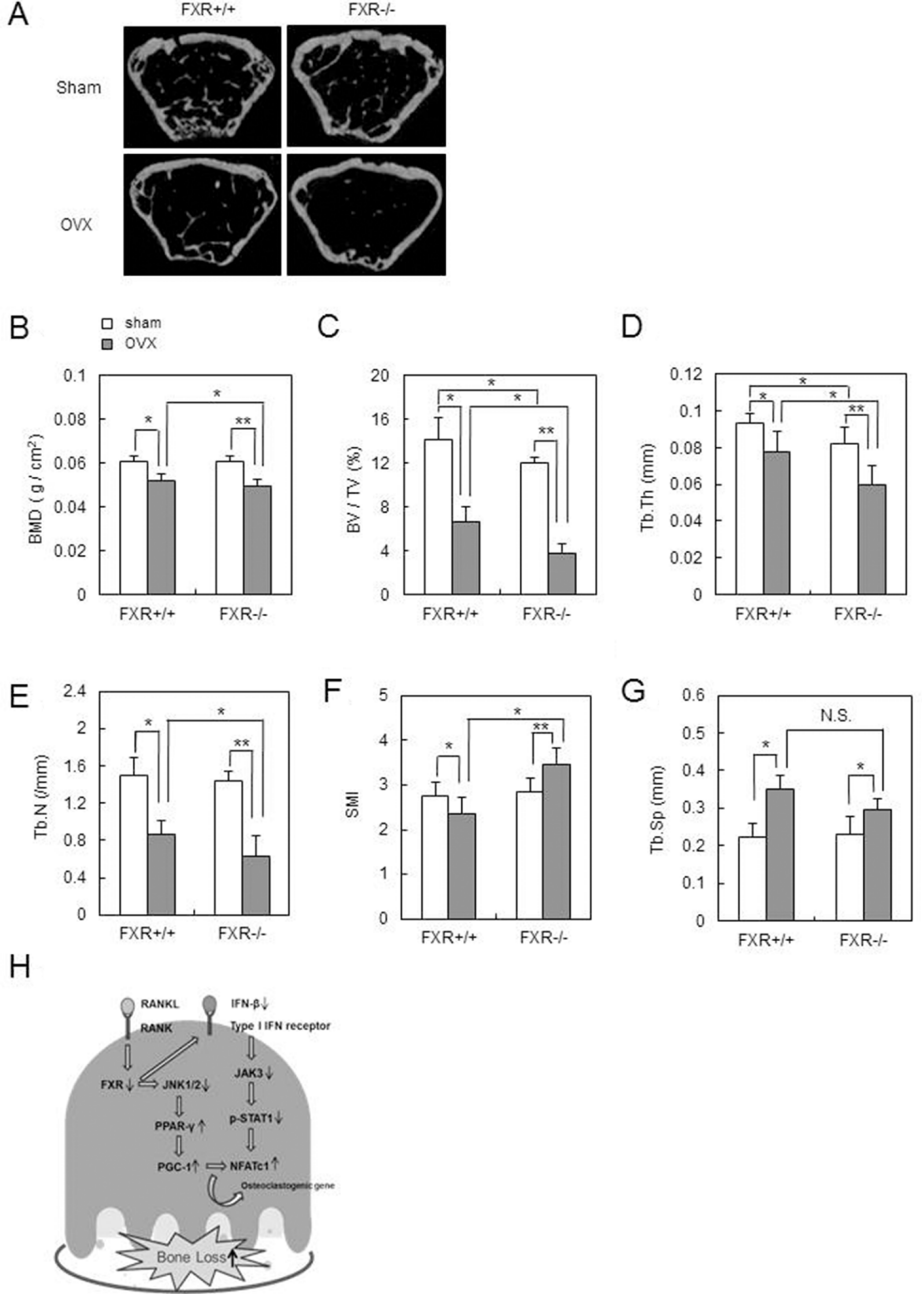
FXR deficiency accelerates OVX-induced bone loss *in vivo* Female 12-week-old mice underwent either OVX or sham operation, and mouse femurs were collected after 10 weeks. (**A**) Two-dimensional micro-CT images of the distal metaphysis of the femurs. (**B**) BMD (g/cm^2^) of femur from FXR^+/+^ and FXR*^−/−^* mice was analyzed by DXA. (**C**–**G**) Various bone parameters of femurs were analyzed by micro-CT. BV/TV (%) (C), Tb.Th (mm) (D), Tb.N (/mm) (E), SMI (F), Tb.Sp (mm) (G). (I) A simplified model for the role of FXR in osteoclastogenesis. *p < 0.05, **p < 0.01. N.S.: Not Significant.

To further investigate the role of FXR in estrogen withdrawal-induced bone loss, we examined FXR^+/+^ and FXR^–/–^ mice that were subjected to sham or OVX *in vivo*. Ten weeks after OVX, bone loss was even more severe in *FXR*^–/–^ mice than in FXR^+/+^ mice (Figure 7A). FXR^–/–^ mice showed lower BMD than FXR^+/+^ mice (Figure 7B). In addition, OVX-induced structural bone alterations, including decreases in BV/TV, Tb.Th, Tb.N, and SMI, were heightened by lack of FXR (Figure 7C–7F). No significant difference was seen in Tb.Sp between FXR^+/+^ and *FXR−/−* mice after OVX (Figure 7G). These data demonstrated that FXR deficiency strengthened the OVX-induced bone loss *in vivo*. Taken together, out data suggest that FXR could be a pivotal modulator of bone loss *in vivo* under pathological conditions.

## DISCUSSION

In this study, we suggest a novel role of FXR, a bile acid-sensing member of the nuclear receptor family, as a negative regulator of RANKL-induced osteoclast differentiation. We found that RANKL down-regulates FXR expression during osteoclast differentiation. Overexpression of FXR in BMMs attenuates RANKL-induced osteoclast differentiation by down-regulation of NFATc1, which is a key modulator for osteoclast differentiation. In agreement with this, *FXR^−/−^* mice exhibited a significantly increased number of osteoclasts, which was partly attributed to a severe osteoporotic phenotype. In an attempt to elucidate the mechanisms by which FXR deficiency increases the osteoclastic differentiation, we demonstrated that the phosphorylation of JNK1/2 by RANKL as well as the expression of JNK1/2 were decreased in *FXR^−/−^ BMMs*. We found that the down-regulation of JNK activity increased the expression of PPARγ and PGC-1β, which might accelerate osteoclast formation in *FXR−/−* BMMs.

The JNKs are members of the MAPK family and are activated by environmental stress [34]. The mammalian JNKs are encoded by three distinct genes (*Jnk1, Jnk2, and Jnk3*). JNK1 and JNK2 isoforms are ubiquitously expressed, whereas JNK3 is almost exclusively found in the brain, heart and testis [34]. Many previous studies have reported the role of JNK1/2 in osteoclast formation. In mouse BMMs, JNK activity is required during osteoclastogenesis to maintain the status of osteoclastic commitment [35]. A study using *JNK1*^−/−^ mice has also reported that activation of JNK1 is required for the efficient osteoclastogenesis of BMMs [36]. However, the present study is distinct from previous genetic and pharmacological studies that investigated the effects of a complete JNK blockade. In the present study, although FXR deficiency failed to effectively induce JNK phosphorylation by RANKL, the basal level of JNK phosphorylation was still maintained in BMMs. We speculate that the maintenance JNK phosphorylation at a basal level permits the acceleration of osteoclastogenesis by increased PPARγ-PGC-1β expression.

There is much evidence to show that the nuclear receptor PPARγ regulates skeletal homeostasis through its direct effect on osteoclasts [25, 30, 37, 38]. Genetically, the loss of PPARγ function in mouse hematopoietic lineages causes osteoclast defects, which present as osteopetrosis [25]. Pharmacologically, rosiglitazone-induced PPARγ activation causes bone loss in mice by enhancing osteoclastogenesis and bone resorption *in vivo* [25]. PPARγ modulates transcription through the ligand-mediated recruitment of coactivators [39]. PGC-1β (also known as Ppargc1β) is a transcriptional coactivator and is strongly induced by RANKL and rosiglitazone during osteoclast differentiation [37, 39]. Knockdown of PGC-1β *in vitro* inhibits osteoclast differentiation, and global PGC-1β deletion in mice results in increased bone mass due to defects in osteoclasts [40]. Furthermore, targeted deletion of PGC-1β in the osteoclast lineage resulted in complete resistance to rosiglitazone-induced bone loss *in vivo* and severe attenuation of rosiglitazone-stimulated osteoclastogenesis *ex vivo* [39]. In accord with these studies, we suggested that the up-regulation of PPARγ and PGC-1β in FXR*^−/−^* BMMs might lead to increased osteoclast formation and bone resorption *in vitro* and *in vivo*. We further demonstrated that the increased expressions of PPARγ and PGC-1β were caused by the reduction of JNK1/2 in FXR*^−/−^* BMMs. Indeed, a previous report demonstrated that PPARγ mRNA levels were increased in JNK1/2-deficient cells, using microarray analysis [41], confirming our present results. To our knowledge, the present study is the first to demonstrate JNK-mediated negative regulation of osteoclastogenesis via PPARγ and PGC-1β.

It has been reported that IFN-β limits the excessive activation of osteoclast differentiation upon RANKL stimulation in a feedback-regulated manner [31]. In fact, *IFN*-β-knock-out mice exhibited an osteoporotic phenotype due to augmented osteoclastogenesis, indicating that IFN-β plays a critical role in the negative regulation of osteoclastogenesis for bone homeostasis [42]. Furthermore, STAT1 has been suggested as a mediator of IFN-β during osteoclastogenesis because STAT1-deficient BMMs are resistant to the suppression of osteoclast differentiation by IFN-β [31, 42]. In this study, it was demonstrated that FXR deficiency impaired the negative regulation of IFN-β signaling in osteoclastogenesis. We also found that FXR acts through JAK3 to modulate IFN-β signaling, since the lack of induction of JAK3 in FXR*^−/−^* BMMs correlated with the resistance to the negative regulatory effects of IFN-β on osteoclastogenesis. These data provide new insights into the role of JAK3 in RANKL-induced osteoclast formation. Taken together, FXR is important to maintain homeostatic osteoclast differentiation through negative regulation of the PPARγ and PGC-1β pathways and positive regulation of the IFN-β and STAT1 pathways.

In addition to the role of FXR in physiological conditions, we also found that FXR modulates osteoclastogenesis and bone loss in pathological conditions caused by unloading or estrogen withdrawal. The world population is aging and the proportion of individuals over 65 is increasing [43]. Considering that menopause occurs around the age of 50, millions of women will live many years at risk for osteoporosis and fractures. Furthermore, in a super-aged society, unloading-induced osteoporosis is also a critical issue in bedridden patients with reduced locomotor function. Thus, an understanding of the molecular mechanisms of menopause- and unloading-induced osteoporosis is required. In this study, our data demonstrated that FXR has potential as a novel drug target for preventing postmenopausal osteoporosis and unloading-induced bone loss.

In summary, we have proposed a new regulatory role of FXR in osteoclastogenesis (Figure 7H) in physiological and pathological bone loss associated with postmenopausal osteoporosis and disuse-induced bone loss. Identification and elucidation of key mediators of RANKL should aid in the development of therapeutic strategies for the treatment of such skeletal diseases.

## MATERIALS AND METHODS

### Reagents

Guggulsterone, SP600125, and rosiglitazone were purchased from Cayman Chemical Company (Ann Arbor, MI, USA). Tofacitinib (JAK3 inhibitor) was purchased from LC Laboratories (Woburn, MA, USA). Antibodies against p-JNK and JNK were purchased from Cell Signaling Technology (Beverly, MA, USA). Antibodies against NFATc1 and JAK3 were purchased from Santa Cruz (Santa Cruz, CA, USA). Recombinant murine M-CSF and RANKL were purchased from Peprotech (Rocky Hill, NJ, USA). All other reagents were purchased from Sigma-Aldrich (St. Louis, MO, USA).

### Animal model

FXR^–/–^ mice were generously provided by F. Gonzalez (NIH, Bethesda, Maryland, USA) and backcrossed to a C57BL/6J background for 10 generations. Genotyping of the mice was performed as previously described [22]. Mouse littermates with a genotype of C57BL/6J WT (FXR*+/+*) were used as controls. All mice were maintained in the animal facility of the Sookmyung Women's University on a 12:12-h light-dark cycle, and were allowed food and water *ad libitum*. All experiments were performed in accordance with the institutional guidelines approved by the Sookmyung Women's University Animal Care and Use Committee.

### Cells and culture system

Mouse bone marrow cells were isolated from 10–12-week-old FXR^+/+^ or FXR*^−/−^* C57BL/6J background mice. Bone marrow cells were cultured in complete α-MEM containing 10% (v/v) fetal bovine serum (FBS), supplemented with 5 ng/ml recombinant murine M-CSF (PeproTech) for 12 h to separate adherent and non-adherent cells. The non-adherent cells were then harvested and cultured with 30 ng/ml M-CSF. After 4 days of culturing, the floating cells were removed and the attached cells were used as BMMs. All cells were cultured in α-MEM containing 5% (v/v) FBS at 37°C in a humidified atmosphere in 5% CO_2_ conditions.

### TRAP staining of osteoclasts

Osteoclasts were observed by staining for TRAP activity. Cultured cells were fixed with 10% formalin for 10 min, permeabilized with ethanol:acetone (50:50 v/v) for 1 min at room temperature, and incubated in acetate buffer (pH 5.2) containing naphthol ASMX phosphate (Sigma-Aldrich) as the substrate and Fast Red Violet LB salt (Sigma-Aldrich) as the dye for the reaction product in the presence of 50 mM sodium tartrate. After washing with distilled water and drying, TRAP^+^ MNCs (*n* > 3) were counted using a light microscope.

### Quantitative real-time PCR

Total RNA was purified with easy-BLUE (iNtRON Biotechnology, Seoul, Korea), and cDNA was prepared from 5 μg of RNA using Revert Aid™ First-Strand cDNA Synthesis Kit (Fermentas, Glen Burnie, MD, USA). Primers for osteoclastogenic genes used in this study are shown in Supplementary Table 1. Real-time PCR reactions were performed in a total volume of 20 μl, using SYBR^®^ Green PCR Master Mix (Applied Biosystems, Foster City, CA, USA) according to the manufacturer's protocol. Thermocycling was performed using a 7500 real-time PCR System (Applied Biosystems) with the following conditions: initial hold at 95°C for 10 min, followed by 40 cycles of denaturation at 95°C for 15 s, annealing at 58°C, and extension at 60°C for 1 min. Data were analyzed using 7500 System Sequence Detection Software version 2.0 (Applied Biosystems). An index mRNA level was assessed using a threshold cycle (Ct) value and normalized against glyceraldehyde 3-phosphate dehydrogenase (GAPDH) expression.

### Immunoblot analysis

Cells were lysed in lysis buffer (50 mM Tris HCl, pH 7.5, 150 mM NaCl, 1% NP-40, 1 mM EDTA, 0.25% SDS, 1 mM NaF, 1 mM Na3VO4, 1 mM phenylmethylsulfonyl fluoride, pepstatin, leupeptin, and aprotinin) and were clarified by centrifugation. Protein was measured by Bradford assay (Bio-Rad Laboratories, Inc., Benicia, CA, USA), and equal amounts of protein were separated by sodium dodecyl sulfate-polyacrylamide gel electrophoresis (SDS-PAGE) and transferred onto a polyvinylidene fluoride (PVDF) membrane (Immobilon-P; Millipore, Bedford, MA, USA). The membranes were blocked with 5% nonfat-milk in phosphate-buffered saline with 0.1% Tween 20 (PBS-T) and then immunostained with the indicated antibodies. The membranes were developed using an enhanced chemiluminescence detection kit (Amersham Biosciences, Buckinghamshire, United Kingdom).

### Bone resorption assay

BMMs were differentiated on dentin slices with M-CSF and RANKL (PeproTech, Inc., Rocky Hill, NJ, USA) for 8 days. The cells were removed from the dentin slice by wiping the its surface, and then the slices were stained with toluidine blue (1 μg/ml; J.T. Baker, Philipsburg, NJ, USA). The number of pits formed by bone resorption on the dentin slices was counted. Image analysis was accomplished using ImageJ software (version 1.32; National Institute of Health, Bethesda, MD, USA) according to the manufacturer's protocol.

### Retroviral gene transduction

To generate retroviral stocks, pMX-IRES-FXR was transfected into the packaging cell line Platinum-E (Plat-E. kindly provided by Prof. S. Y. Lee at Ewha University). Viral supernatant was collected from culture media at 48 h after transfection using Lipofectamine 2000 (Invitrogen, Carlsbad, CA, USA) according to the manufacturer's instructions. For infection with retroviruses, BMMs were incubated with the viral supernatant (4 ml/dish), polybrene (10 μg/ml, Santa Cruz, CA, USA), and M-CSF (30 ng/ml) for 2 days, and selected by puromycin (2 μg/ml; Sigma-Aldrich, St. Louis, MO, USA) for an additional 48 h.

### siRNA transfection

BMMs were seeded into 48-well plates at a density of 3.5 × 10^4^ cells/well with 30 ng/ml M-CSF. After 24 h, cells were transfected with 40 nM mouse JNK on-target plus smart pool siRNAs (5′-AAAGAATGTCCTACCTTCT-3′) (QIAGEN, Hilden, Germany) or mouse JAK3 on-target plus smart pool siRNAs (5′-CACGTTAGACTTTGCCATCCA-3′; QIAGEN) using Lipofectamine 2000 (Invitrogen, Carlsbad, CA, USA) according to the manufacturer's instructions. The control contained 40 nM non-targeting siRNA (QIAGEN). The transfection took place in 2.5 ml of serum-free medium for 6 h; the cells were then cultured for 4 days in complete media containing 30 ng/ml M-CSF and 100 ng/ml RANKL for osteoclast formation.

### LPS-induced osteoclast formation *in vivo*

To investigate the contribution of FXR to LPS-induced osteoclast formation *in vivo*, 12-week-old FXR^+/+^ and FXR*−/−* male mice were directly injected with 0.5 mg/mouse LPS on calvarial bone (*n* = 5 per each group). After 6 days, mice were euthanized. Whole calvaria were fixed in 4% paraformaldehyde for 24 h and then stained with TRAP. Image analysis was accomplished using ImageJ software (version 1.32; National Institute of Health, Bethesda, MD, USA) according to the manufacturer's protocol.

### Rosiglitazone-induced bone loss *in vivo*

12-week-old male FXR^+/+^ and FXR*^−/−^* mice were orally administered daily either with rosiglitazone (10 mg/kg; Cayman Chemical Company, Ann Arbor, MI, USA) or with control (*n* = 10 per each group). The mice were euthanized 2 months after administration. Femurs of mice were fixed with 70% ethanol at 4°C until analysis. Bone mineral density (BMD; g/cm^2^) of femurs was measured and analyzed by the dual energy X-ray absorptiometry (DXA) instrument PIXIMUS (GE Lunar, Madison, WI, USA). Three-dimensional measurements were performed with a micro-CT scanner and associated analysis software (Model 1172; Skyscan, Antwerp, Belgium) at 9-mm voxel size. Image acquisition was performed at 35 kV of energy and at an intensity of 220 mA. The threshold was set to segment the bone from the background, and the same threshold setting was used for all samples.

### Tail suspension model

Tail suspension was conducted by applying a tape to the surface of the hindlimb to set a metal clip [23]. The other end of the clip was fixed to an overhead bar. The height of the bar was adjusted to maintain the mice at a 30 degrees head-down tilt with the hindlimbs elevated above the floor of the cage. Male 8-week-old FXR^+/+^ and FXR*^−/−^* mice were subjected to tail suspension for 7 days. Loaded control mice were also housed under the same conditions (except for tail suspension) and for the same duration (*n* = 7 per group).

### OVX-induced bone loss *in vivo*

Female 12-week-old FXR^+/+^ and FXR*^−/−^* mice underwent either ovariectomy (OVX) or sham operation (*n* = 14 per group). The mice were euthanized after 10 weeks. Femurs of mice were fixed with 70% ethanol at 4°C and were analyzed by DXA and micro-CT as mentioned above.

### Statistical analysis

The descriptive statistics present data as the mean ± SD. Means were compared by Student's *t*-test (for comparison of two means) or ANOVA (for multiple comparisons) with a least significant difference post hoc test. Data represent the means and the SD from at least three independent experiments. Data were reported as statistically significant when the *p*-value < 0.05 for comparisons. Calculations were performed with the software package SPSS (Ver. 21.0 for windows; SPSS, Inc., Chicago, IL, USA).

## SUPPLEMENTARY MATERIALS FIGURES AND TABLES


