# Supplementary material for: The farnesoid X receptor negatively regulates osteoclastogenesis in bone remodeling and pathological bone loss
Source: Oncotarget. 2017 Aug 28;8(44):76558–73. doi: 10.18632/oncotarget.20576 (PMC5652726; doi:10.18632/oncotarget.20576)
Supplement: Supplementary file 1 [file oncotarget-08-76558-s001.pdf]

# The farnesoid X receptor negatively regulates osteoclastogenesis in bone remodeling and pathological bone loss

## SUPPLEMENTARY MATERIALS

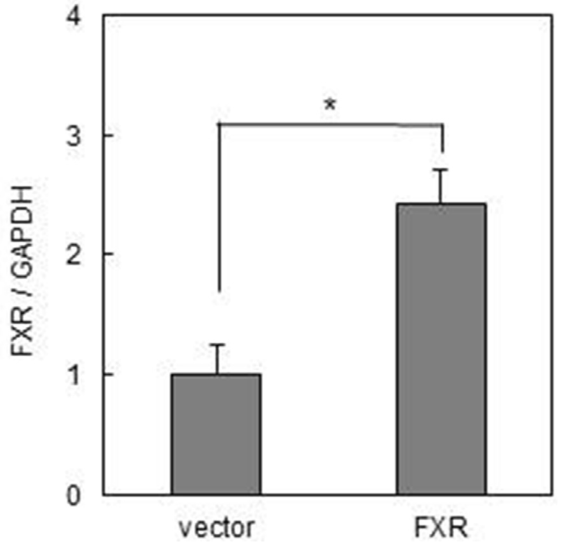


**Supplementary Figure 1: Overexpression of farnesoid X receptor (FXR) in FXR+/+ bone marrow-derived macrophages (BMMs).** BMMs were infected through a retrovirus packaging system. The mRNA expression of FXR in infected BMMs was analyzed by real-time PCR. * $p < 0.05$

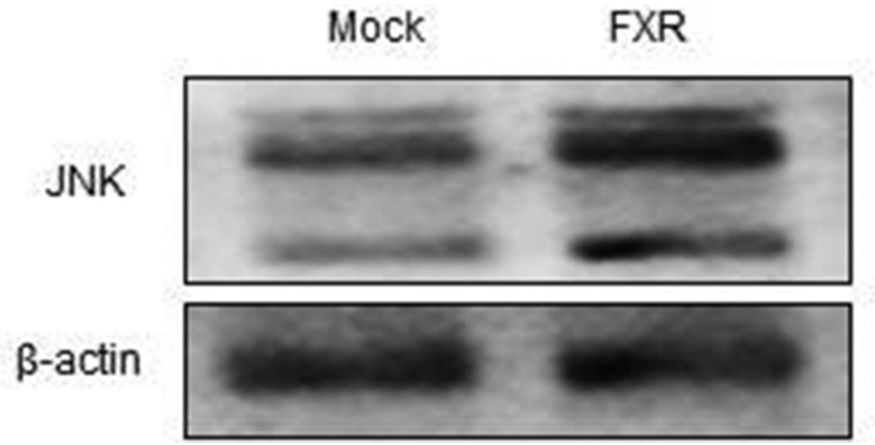


**Supplementary Figure 2: Modulation of JNK1/2 expression levels by overexpression of FXR.** BMMs were infected by mock or FXR through a retrovirus packaging system. The protein expression of JNK1/2 in infected BMMs was analyzed by western blotting.

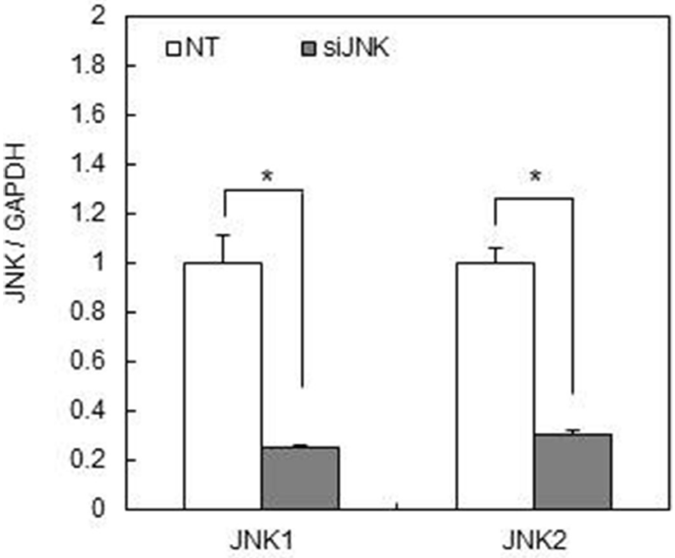


**Supplementary Figure 3: The JNK1/2 mRNA levels by knockdown of JNK.** The mRNA expression level of JNK1/2 in siRNA-transfected BMMs was analyzed by real-time PCR. * $p < 0.05$

## Supplementary Table 1: Mouse genes primers used for real-time PCR analysis

| Mouse genes | primer sequences |
|---|---|
| FXR | 5′-TGT GAG GGC TGC AAA GGT TT-3′ |
| | 5′-ACA TCC CCA TCT CTC TGC AC-3′ |
| JNK1 | 5′-TGC CAT CAT GAG CAG AAG CAA ACG-3′ |
| | 5′-TCT GAT TCT GAA ATG GCC GGC TGA-3′ |
| JNK2 | 5′-GTG GCG GAC TCA ACT TTC ACT GTT-3′ |
| | 5′-TCT CTT TGC GTG CGT TTG GTT CTG-3′ |
| PPARγ | 5′-TGC TGT TAT GGG TGA AAC TCT GGG-3′ |
| | 5′-GGCTTGATGTCAAAGGAATGCG-3′ |
| PGC-1β | 5′-TACATGCATACCTACTGCCTGCCT-3′ |
| | 5′-TTGGGCCAGAAGTTCCCTTAGGAT-3′ |
| JAK3 | 5'-TTA CAA AGCCTGTCTGCCG-3′ |
| | 5'-TCCAGGAGATGCCGTTGTC-3' |
| IFN-β | 5'-CTGGCTTCCATCATGAACAA-3' |
| | 5'-CATTTCCGAATGTTCGTCCT-3′ |
| GAPDH | 5′-TGCACCACCAACTGCTTATTAGC-3′ |
| | 5′-GGCATGGACTGTGGTCATGAG-3′ |
